# Concomitant epigenetic targeting of LSD1 and HDAC synergistically induces mitochondrial apoptosis in rhabdomyosarcoma cells

**DOI:** 10.1038/cddis.2017.239

**Published:** 2017-06-15

**Authors:** Tinka Haydn, Eric Metzger, Roland Schuele, Simone Fulda

**Affiliations:** 1 Institute for Experimental Cancer Research in Pediatrics, Goethe-University, Komturstrasse 3a, Frankfurt 60528, Germany; 2 German Cancer Consortium (DKTK), Partner Site Frankfurt, Germany; 3 German Cancer Research Center (DKFZ), Heidelberg, Germany; 4 Department of Urology, University Freiburg Medical Center, Freiburg, Germany

## Abstract

The lysine-specific demethylase 1 (LSD1) is overexpressed in several cancers including rhabdomyosarcoma (RMS). However, little is yet known about whether or not LSD1 may serve as therapeutic target in RMS. We therefore investigated the potential of LSD1 inhibitors alone or in combination with other epigenetic modifiers such as histone deacetylase (HDAC) inhibitors. Here, we identify a synergistic interaction of LSD1 inhibitors (i.e., GSK690, Ex917) and HDAC inhibitors (i.e., JNJ-26481585, SAHA) to induce cell death in RMS cells. By comparison, LSD1 inhibitors as single agents exhibit little cytotoxicity against RMS cells. Mechanistically, GSK690 acts in concert with JNJ-26481585 to upregulate mRNA levels of the proapoptotic BH3-only proteins BMF, PUMA, BIM and NOXA. This increase in mRNA levels is accompanied by a corresponding upregulation of BMF, PUMA, BIM and NOXA protein levels. Importantly, individual knockdown of either BMF, BIM or NOXA significantly reduces GSK690/JNJ-26481585-mediated cell death. Similarly, genetic silencing of BAK significantly rescues cell death upon GSK690/JNJ-26481585 cotreatment. Also, overexpression of antiapoptotic BCL-2 or MCL-1 significantly protects RMS cells from GSK690/JNJ-26481585-induced cell death. Furthermore, GSK690 acts in concert with JNJ-26481585 to increase activation of caspase-9 and -3. Consistently, addition of the pan-caspase inhibitor N-benzyloxycarbonyl-Val-Ala-Asp-fluoromethylketone (zVAD.fmk) significantly reduces GSK690/JNJ-26481585-mediated cell death. In conclusion, concomitant LSD1 and HDAC inhibition synergistically induces cell death in RMS cells by shifting the ratio of pro- and antiapoptotic BCL-2 proteins in favor of apoptosis, thereby engaging the intrinsic apoptotic pathway. This indicates that combined treatment with LSD1 and HDAC inhibitors is a promising new therapeutic approach in RMS.

RMS represents the most frequent soft-tissue sarcoma in children and comprises two major subtypes, that is, embryonal RMS (eRMS) and alveolar RMS (aRMS).^1, 2, 3^ Despite multimodal therapy consisting of surgery, chemotherapy and radiation, the overall survival for patients with advanced disease is still very poor.^4^ This highlights the urgent medical need for innovative treatment concepts.

The antineoplastic activity of chemo-, immuno-, or radiotherapy largely depends on the induction of programmed cell death in tumor cells.^5^ Apoptosis is one of the most extensively studied forms of programmed cell death that is highly conserved throughout evolution and typically disturbed in cancer cells.^6^ Two key signaling pathways to apoptotic cell death have been delineated, namely the intrinsic (mitochondrial) and the extrinsic (death-receptor) pathway, which both eventually lead to activation of caspases.^5, 7^ Within the intrinsic pathway, pro- and antiapoptotic proteins of the BCL-2 family control outer mitochondrial membrane permeabilization (MOMP).^7, 8^ A shift towards proapoptotic BCL-2 family proteins favors MOMP, followed by the release of cytochrome C and second mitochondria-derived activator of caspases (Smac) from the mitochondrial intermembrane space into the cytosol.^7, 8^ Cytochrome C initiates formation of the apoptosome and activation of initiator caspase-9 which in turn activates caspase-3, eventually leading to the execution of apoptotic cell death.^9^ Smac contributes to the activation of caspases as it binds to and thereby antagonizes XIAP, a member of the Inhibitor of Apoptosis family of proteins.^10^

Post-translational modifications of histone proteins such as acetylation, methylation or phosphorylation create a histone code, which provides the basis for the transcriptional activity of numerous genes.^11, 12^ Removal of histone acetylation and demethylation of H3K4 reduce transcriptional activity and are conducted by repressor complexes, like the CoREST complex that contains HDAC1 or HDAC2, as well as LSD1.^13, 14, 15, 16^ HDACs have been implicated in contributing to oncogenesis by silencing tumor suppressor genes and apoptosis inducers.^17, 18^ LSD1 is known as a regulator of a wide spectrum of biological processes including pluripotency, differentiation, metabolic processes, as well as cancer development and progression.^19, 20, 21^ In RMS, HDAC inhibition has been shown to reverse oncogenic features and induce cell death.^22, 23, 24^ In recent years, a broad range of inhibitors of epigenetic modifiers has been developed. JNJ-26481585 (Quisinostat) is a second-generation HDAC inhibitor that blocks class I and II HDACs with high potency.^25^

LSD1 inhibition was first described for the antidepressant agent Tranylcypromine, a MAO-A and MAO-B inhibitor that also inhibits LSD1 due to the high similarity of the catalytic sites of LSD1, MAO-A and MAO-B.^26^ In recent years, more specific LSD1 inhibitors have been developed, some of which have already progressed to clinical trials for the treatment of leukemia or lung cancer.^27, 28^ High LSD1 levels have been detected in several types of solid tumors or hematological malignancies and have been associated with poor prognosis.^19^ Recently, LSD1 has also been shown to be overexpressed in primary RMS samples.^29, 30^ However, little is yet known about whether or not LSD1 may serve as a therapeutic target in RMS. Therefore, the current study aims at investigating the potential of LSD1 inhibition in RMS cells, either alone or in combination with other epigenetic modifiers such as HDAC inhibitors.

## Results

### LSD1 and HDAC inhibitors synergize to induce cell death in RMS cells

**Figure 1:**
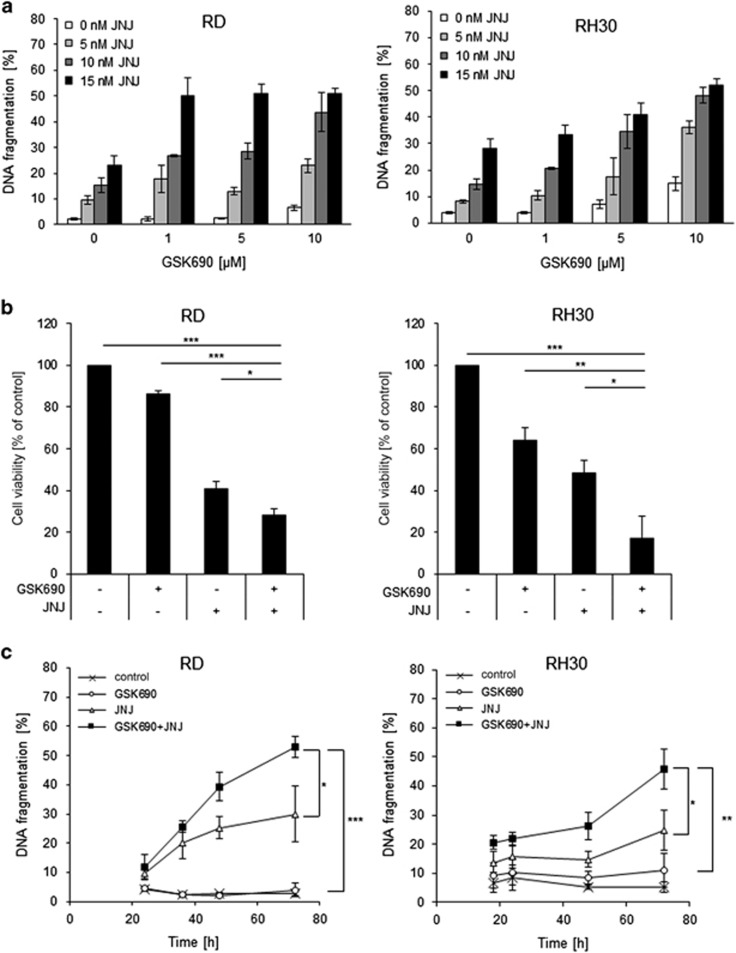
LSD1 and HDAC inhibitors synergize to induce cell death in RMS cells. (**a**) Cells were treated with indicated concentrations of GSK690 and/or JNJ-26481585 for 72 h. Cell death was determined by flow cytometric analysis of DNA fragmentation of PI-stained nuclei. (**b**) Cells were treated with 1 *μ*M GSK690 and 15 nM JNJ-26481585 (RD) or 10 *μ*M GSK690 and 5 nM JNJ-26481585 (RH30) for 72 h. Cell viability was assessed by MTT assay. (**c**) Cells were treated with 1 *μ*M GSK690 (RD cells) or 10 *μ*M GSK690 (RH30 cells) and/or 15 nM JNJ-26481585 for indicated time points. Cell death was determined by flow cytometric analysis of DNA fragmentation of PI-stained nuclei. In (**a-c**) mean and S.D. of three independent experiments performed in triplicate are shown; *P<0.05; **P<0.01; ***P<0.001

To investigate the therapeutic potential of LSD1 inhibition in RMS, we tested the effects of the reversible LSD1 inhibitor GSK690 alone and in combination with the second-generation HDAC inhibitor JNJ-26481585 in RMS cell lines, which represent eRMS (RD, TE381.T) and aRMS (RH30, RMS13) as the two major histological subtypes. Treatment with GSK690 alone had no or little effect on the induction of cell death, as measured by DNA fragmentation, a typical parameter of apoptotic cell death (Figure 1a), and by propidium iodide (PI) staining (Supplementary Fig. 1A). Importantly, GSK690 acted together with JNJ-26481585 to induce cell death in all four tested RMS cells lines (Figure 1a,Supplementary Fig. 1A, 1B). Calculation of combination index (CI) revealed that the interaction of both agents is synergistic (Supplementary Table 1). To investigate whether this synergy extends to other LSD1 and HDAC inhibitors, we used the LSD1 inhibitor Ex917 and the HDAC inhibitor SAHA. Similarly, Ex917 plus JNJ-26481585 as well as SAHA plus GSK690 acted in concert to trigger cell death, whereas treatment with LSD1 inhibitors alone induced basically no cell death (Supplementary Fig. 1C, 1D). Also, GSK690 and JNJ-26481585 cooperated to reduce cell viability (Figure 1b). Assessment after 120 h of exposure to GSK690 and JNJ-26481585 showed profound suppression of cell viability over prolonged time (Supplementary Fig. 1E). Treatment with Ex917 alone at the tested concentrations did not affect cell viability and GSK690 decreased cell viability only at high concentrations (Supplementary Fig. 2A, 2B). Furthermore, the addition of GSK690 further enhanced the JNJ-26481585-stimulated G2/M arrest (Supplementary Fig. 3).

To assess whether inhibition of LSD1 alters sensitivity of non-malignant cells to HDAC inhibitors we extended our experiments to the murine myoblast cell line C2C12. Treatment with different combinations of LSD1 and HDAC inhibitors at the same concentrations used for RMS cells (i.e., GSK690/JNJ-26481585, Ex917/JNJ-26481585 or GSK690/SAHA) induced no or little (< 20%) cell death in C2C12 cells (Supplementary Fig. 4A–C), thus pointing to some tumor selectivity. Monitoring the kinetics of cell death induction showed that GSK690 significantly increased JNJ-26481585-induced cell death in a time-dependent manner (Figure 1c). Control experiments confirmed that LSD1 and HDAC inhibitors caused their typical target-specific effects on histone-modifying enzymes, that is, increased H3K4me2 by LSD1 inhibitors and enhanced histone acetylation by HDAC inhibitors (Supplementary Fig. 5A, 5B). At an early time point, the addition of GSK690 enhanced the JNJ-26481585-stimulated acetylation of histone H3 (Supplementary Fig. 5C), indicating that both drugs cooperate at the histone level. Together, this set of experiments demonstrates that LSD1 and HDAC inhibitors synergize to induce cell death in RMS cells.

### GSK690/JNJ-26481585 cotreatment induces caspase-dependent cell death

**Figure 2:**
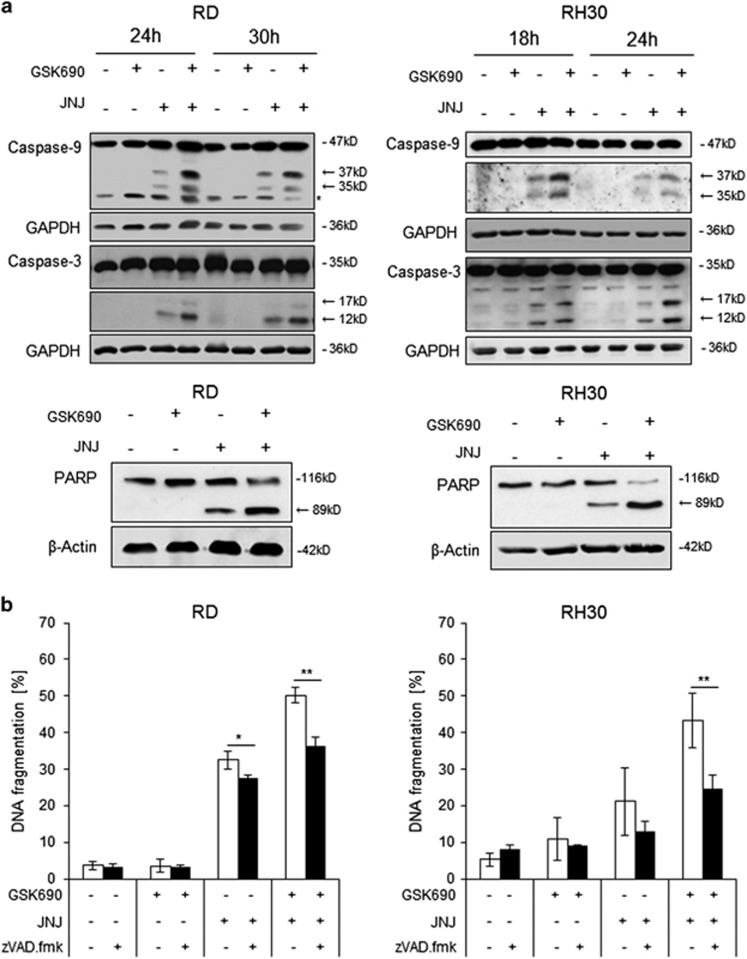
GSK690/JNJ-26481585 cotreatment induces caspase-dependent cell death. (**a**) Cells were treated for indicated times with 1 *μ*M GSK690 (RD cells) or 10 *μ*M GSK690 (RH30 cells) and/or 15 nM JNJ-26481585. Caspase-3 and caspase-9 cleavage was detected by Western blotting, cleavage products are indicated by arrows. GAPDH was used as loading control. Cells were treated with 1 *μ*M GSK690 (RD cells) or 10 *μ*M GSK690 (RH30 cells) and/or 15 nM JNJ-26481585 for 21 h (RD cells) or 15 h (RH30 cells). PARP cleavage was detected by Western blotting, the cleavage product is indicated by arrow; β-Actin was used as loading control. (**b**) Cells were treated for 72 h with 1 *μ*M GSK690 (RD cells) or 10 *μ*M GSK690 (RH30 cells) and/or 15 nM JNJ-26481585 in the presence or absence of 50 *μ*M zVAD.fmk. Cell death was determined by flow cytometric analysis of DNA fragmentation of PI-stained nuclei. Mean and SD of three independent experiments performed in triplicate are shown; *P<0.05; **P<0.01

To gain insights into the molecular mechanisms of apoptotic cell death upon treatment with GSK690 and JNJ-26481585, we investigated caspase cleavage as an indicator of caspase activation. GSK690 and JNJ-26481585 cooperated to induce cleavage of the initiator caspase-9 into its active p35 and p37 fragments and of the effector caspase-3 into its active p12 and p17 fragments (Figure 2a). Moreover, GSK690 and JNJ-26481585 acted together to cleave poly ADP-ribose polymerase (PARP), a known substrate of caspase-3 (Figure 2a). To investigate whether caspase activity is necessary for cell death induction, we used the pan-caspase inhibitor zVAD.fmk. Addition of zVAD.fmk significantly reduced GSK690/JNJ-26481585-induced cell death (Figure 2b). A kinetic analysis showed that zVAD.fmk almost completely rescued GSK690/JNJ-26481585-conferred cell death at earlier time points in RH30 cells and also significantly protected RD cells at all tested time points (Supplementary. Fig. 6A). Furthermore, we used the RIP1 kinase inhibitor Necrostatin-1 to explore whether RIP1 is required for cell death. However, addition of Necrostatin-1 failed to rescue cells from GSK690/JNJ-26481585-induced cell death (Supplementary Fig. 6B). Altogether, these findings demonstrate that GSK690/JNJ-26481585 cotreatment induces caspase-dependent cell death.

### GSK690/JNJ-26481585 cotreatment alters the balance between pro- and antiapoptotic proteins

**Figure 3:**
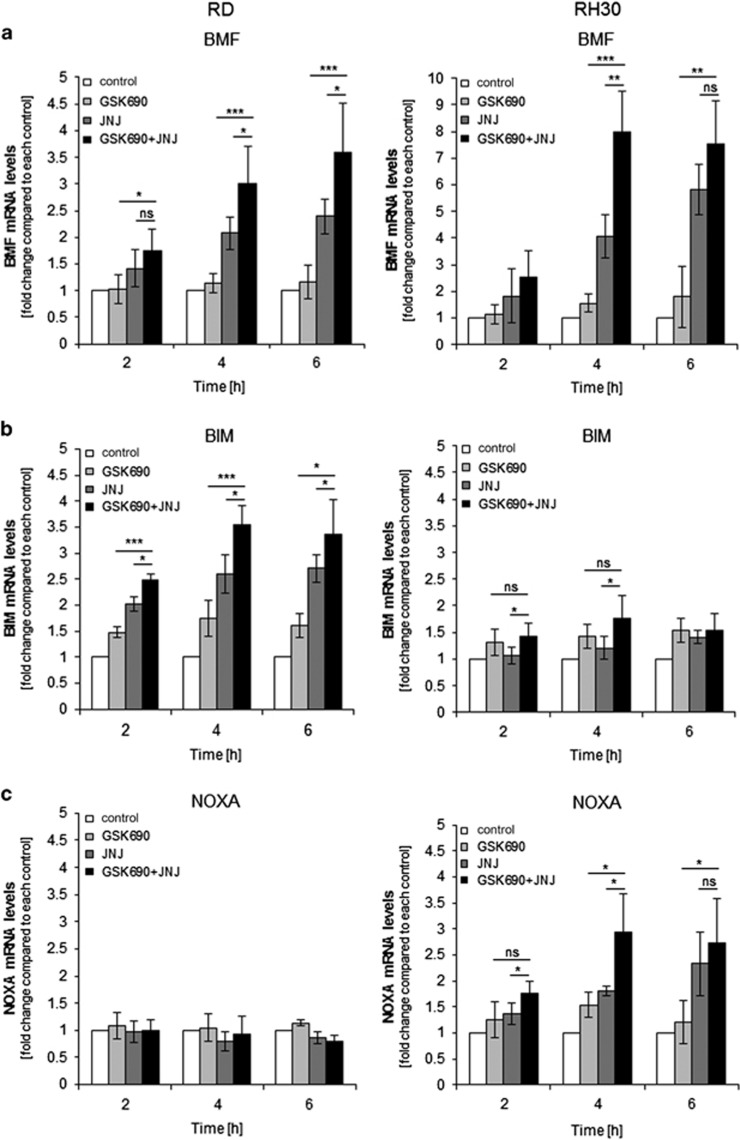

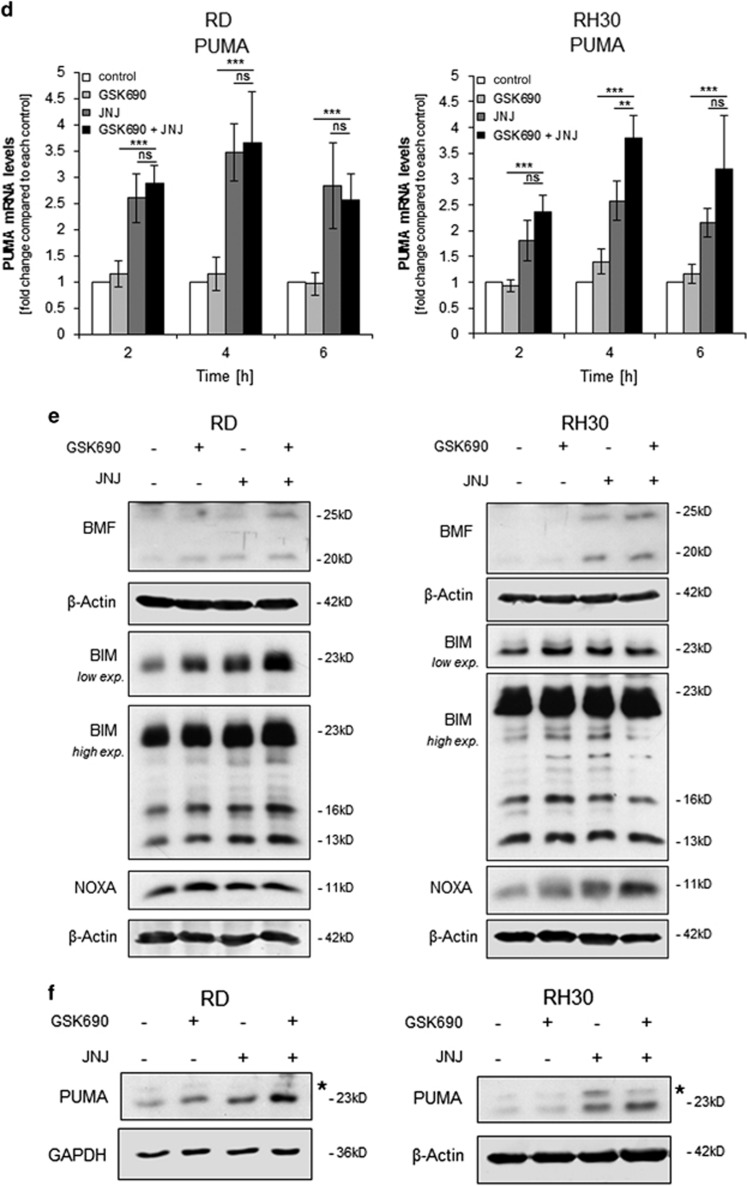
GSK690/JNJ-26481585 cotreatment alters the balance between pro- and antiapoptotic proteins. (**a-d**) Cells were treated for indicated times with 1 *μ*M GSK690 (RD cells) or 10 *μ*M GSK690 (RH30 cells) and/or 15 nM JNJ-26481585. Expression levels of BMF, BIM, NOXA, and PUMA mRNA were analyzed by qRT-PCR and fold changes relative to untreated control of each time point are shown with mean and S.D. of three independent experiments performed in duplicate; *P<0.05; **P<0.01; ***P<0.001; ns, not significant. (**e**) Cells were treated for 6 h with 1 *μ*M GSK690 (RD cells) or 10 *μ*M GSK690 (RH30 cells) and/or 15 nM JNJ-26481585. Protein levels of BMF (20 and 25 kDa), BIM and NOXA were detected by Western blotting. β-Actin was used as loading control. (**f**) Cells were treated for 9 h with 1 *μ*M GSK690 (RD cells) or 10 *μ*M GSK690 (RH30 cells) and/or 15 nM JNJ-26481585. Protein levels of PUMA were detected by Western blotting. GAPDH and β-Actin were used as loading controls. Asterisks indicate unspecific bands

As both GSK690 and JNJ-26481585 can alter histone modifications towards more active transcriptional sites, we next explored whether GSK690/JNJ-26481585 cotreatment alters mRNA expression of proapoptotic BCL-2 family proteins. Importantly, GSK690/JNJ-26481585 cotreatment stimulated upregulation of BMF and PUMA mRNA levels in both cell lines (Figures 3a and d) prior to the onset of cell death (Figure 1c). In addition, GSK690 cooperated with JNJ-26481585 to increase BIM mRNA levels in RD cells and NOXA mRNA levels in RH30 cells (Figures 3b and c).

To determine whether these changes in mRNA levels result in elevated protein expression, we performed Western blotting. Notably, combination of GSK690 plus JNJ-26481585 upregulated BMF and PUMA protein in both cell lines (Figures 3e and f). Also, the combination treatment enhanced BIM expression in RD cells and NOXA expression in RH30 cells (Figure 3e), corresponding to the GSK690/JNJ-26481585-stimulated increase in their mRNA levels (Figures 3a–c). It is interesting to note that NOXA was upregulated by GSK690/JNJ-26481585 treatment in RH30 cells, which harbor much lower basal NOXA protein levels than RD cells, while BIM expression was increased by GSK690/JNJ-26481585 treatment in RD cells that exhibit lower constitutive BIM protein levels compared to RH30 cells (Supplementary Fig. 7A). By comparison, protein levels of BCL-2, BCL-x_L_, and MCL-1 remained largely unchanged upon GSK690/JNJ-26481585 cotreatment (Supplementary Fig. 7B).

Together, this set of experiments shows that prior to the onset of cell death GSK690/JNJ-26481585 cotreatment upregulates proapoptotic BH3-only proteins and thus changes the ratio of pro- and antiapoptotic BCL-2 family proteins towards apoptosis.

### BMF, BIM, and NOXA contribute to GSK690/JNJ-26481585-induced apoptosis

**Figure 4:**
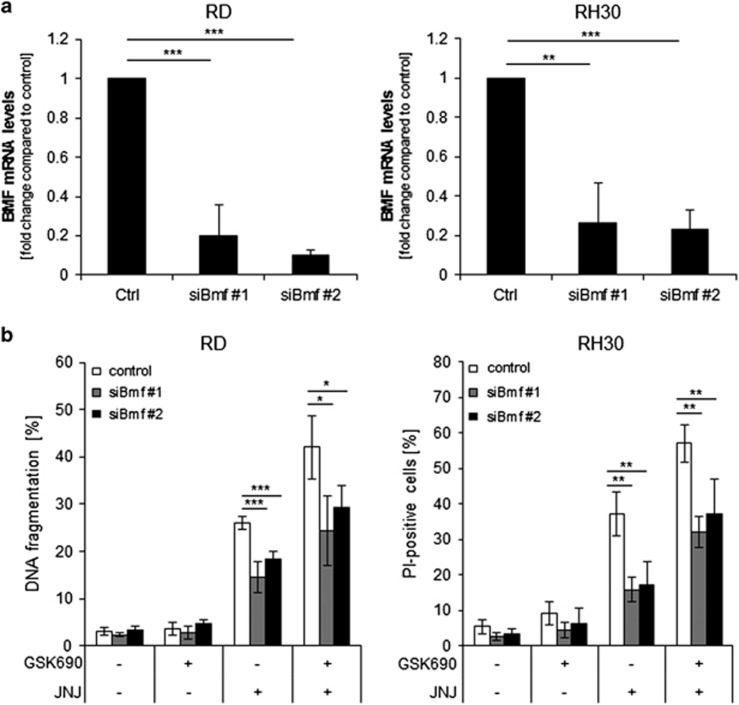

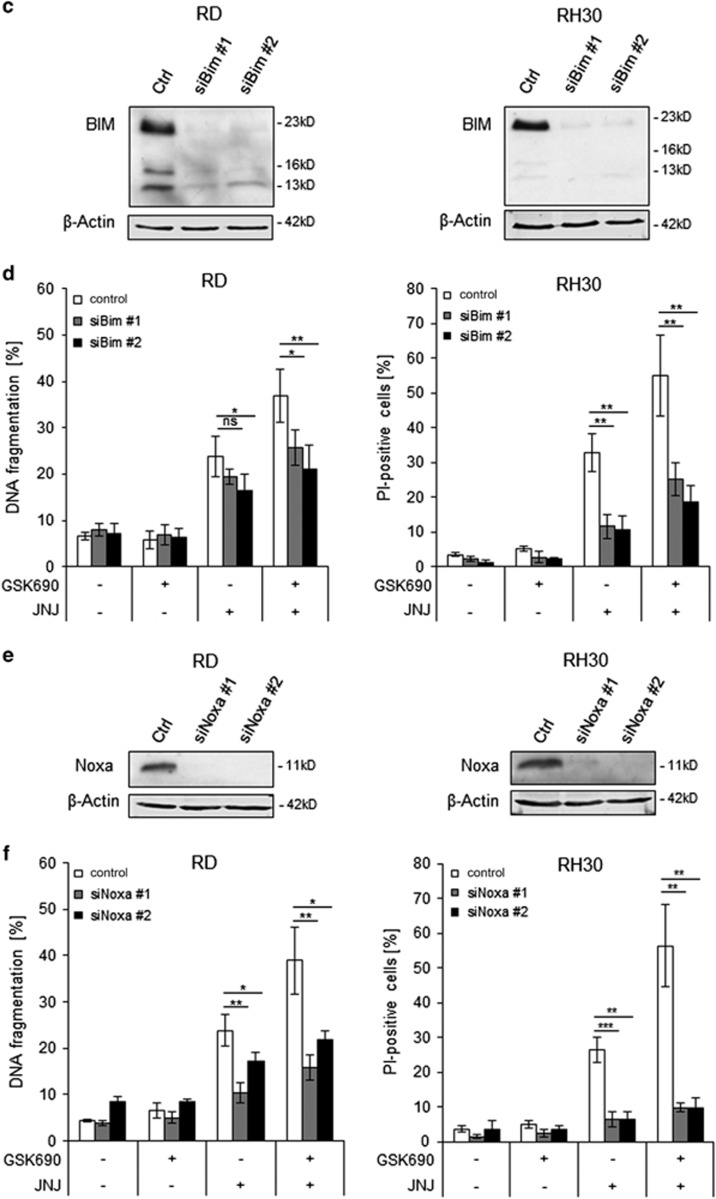
BMF, BIM and NOXA contribute to GSK690/JNJ-26481585-induced apoptosis. RD and RH30 were transiently transfected for 24 h with non-silencing siRNA or siRNA targeting BMF, BIM or NOXA mRNA. (**a, c,e**), BMF mRNA levels were assessed by qRT-PCR (**a**), BIM (**c**) and NOXA (**e**) protein levels were detected by Western blotting; β-Actin was used as loading control. (**b, d,f**), Cells were treated 24 h after transfection with 1 *μ*M GSK690 (RD cells) or 5 *μ*M GSK690 (RH30 cells) and/or 10 nM JNJ-26481585 for 72 h. Cell death was determined by flow cytometric analysis of DNA fragmentation of PI-stained nuclei (RD cells) or fluorescence-based microscope analysis of PI uptake using Hoechst 33342 and PI double staining (RH30 cells). In (**b, d,f**), mean and SD of three independent experiments performed in triplicate are shown; *P<0.05; **P<0.01; ***P<0.001

To test the functional relevance of the observed changes in proapoptotic BH3-only proteins for the induction of apoptosis, we individually silenced BMF, BIM, or NOXA using two distinct siRNA sequences. Control experiments confirmed efficient knockdown of target genes (Figures 4a, c, e). Importantly, knockdown of either BMF, BIM or NOXA significantly rescued both RD and RH30 cells from GSK690/JNJ-26481585-triggered cell death (Figures 4b, d, f). These findings indicate that BMF, BIM and NOXA contribute to GSK690/JNJ-26481585-mediated cell death.

### BAK contributes to GSK690/JNJ-26481585-induced apoptosis

**Figure 5:**
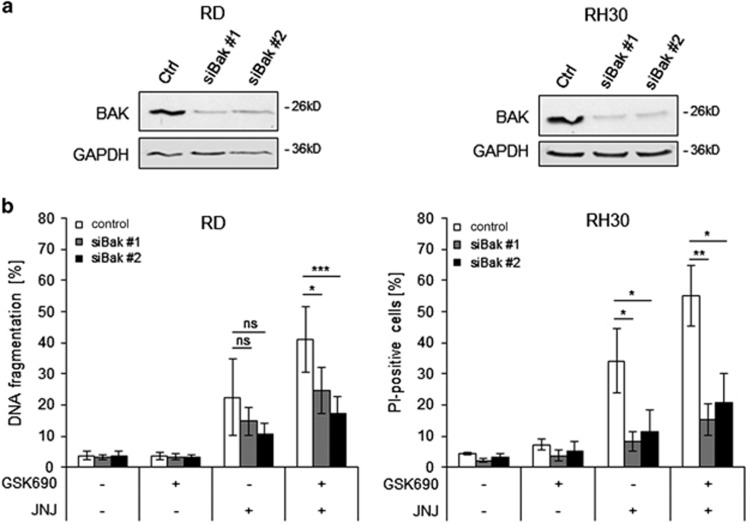
BAK contributes to GSK690/JNJ-26481585-induced apoptosis. RD and RH30 were transiently transfected for 48 h with non-silencing siRNA or siRNA targeting BAK. (**a**) Knockdown of BAK protein was detected by Western blotting, GAPDH served as loading control. (**b**) Cells were treated 48 h after transfection with 1 *μ*M GSK690 (RD cells) or 10*μ*M GSK690 (RH30 cells) and/or 15 nM JNJ-26481585 for 72 h and cell death was determined by flow cytometric analysis of DNA fragmentation of PI-stained nuclei (RD cells) or fluorescence-based microscope analysis of PI uptake using Hoechst 33342 and PI double staining (RH30 cells). Mean and S.D. of three independent experiments performed in triplicate are shown; *P<0.05; **P<0.01; ***P<0.001

As BAK, a multi-domain proapoptotic BCL-2 family protein, plays an important role in mediating mitochondrial apoptosis,^31^ we next silenced BAK to test the requirement of an intact intrinsic pathway. Western blotting confirmed efficient silencing of BAK by two distinct siRNA sequences (Figure 5a). Notably, knockdown of BAK significantly rescued both RMS cell lines from GSK690/JNJ-26481585-induced cell death (Figure 5b). This shows that BAK contributes to GSK690/JNJ-26481585-induced apoptosis.

### Overexpression of BCL-2 or MCL-1 reduces GSK690/JNJ-26481585-induced apoptosis

**Figure 6:**
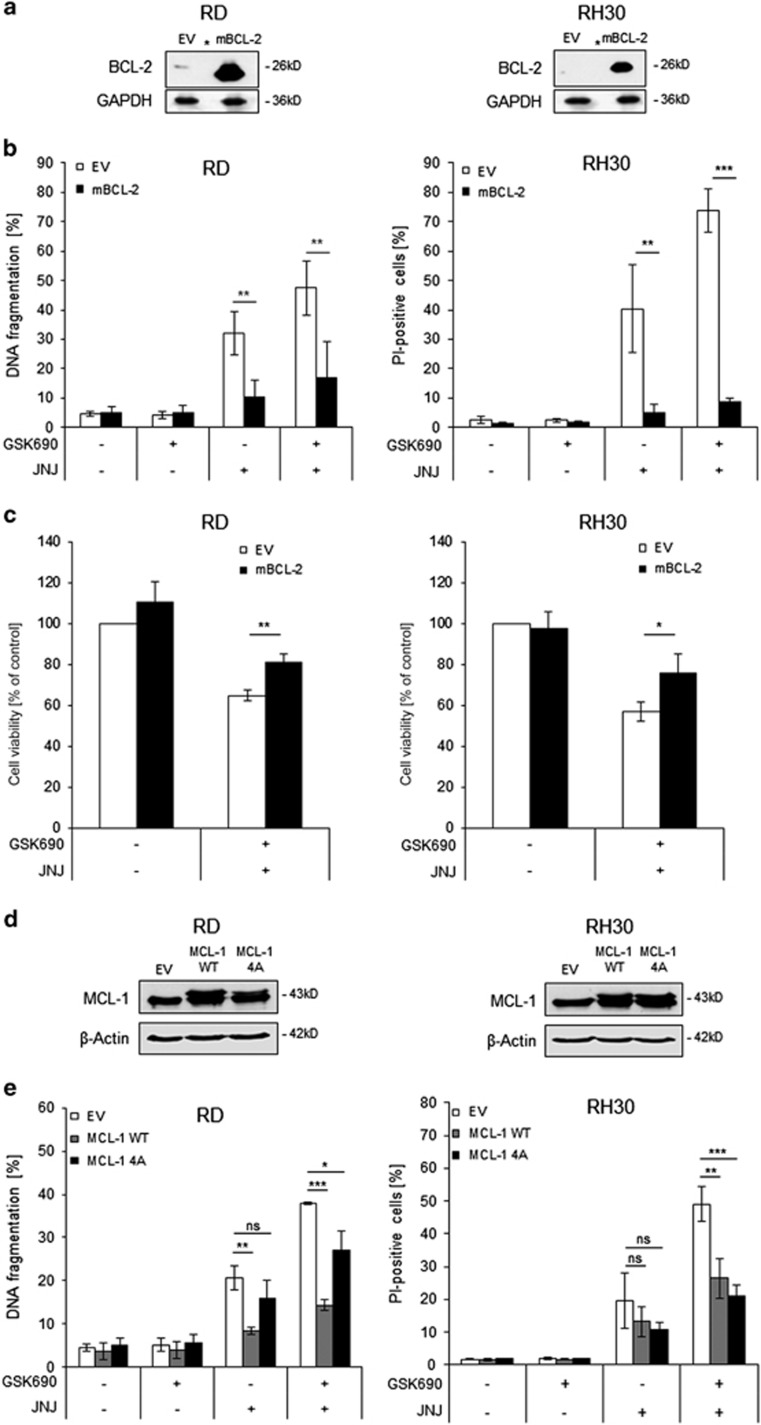
Overexpression of BCL-2 or MCL-1 reduces GSK690/JNJ-26481585-induced apoptosis. (**a**) RD and RH30 were transfected with empty vector (EV) or a vector containing a murine BCL-2 construct (mBCL-2). Expression of BCL-2 was assessed by Western blotting, asterisks indicate empty lanes. GAPDH was used as loading control. (**b**) Cells were treated with 1 *μ*M GSK690 (RD cells) or 5 *μ*M GSK690 (RH30 cells) and/or 15 nM JNJ-26481585 for 72 h. Cell death was determined by flow cytometric analysis of DNA fragmentation of PI-stained nuclei (RD cells) or fluorescence-based microscope analysis of PI uptake using Hoechst 33342 and PI double staining (RH30 cells). (**c**) Cells were treated with 1 *μ*M GSK690 (RD cells) or 10 *μ*M GSK690 (RH30 cells) and/or 15 nM JNJ-26481585 for 36 h (RD) or 24 h (RH30). Cell viability was assessed with MTT assay. (**d**) Cells were transfected with EV, wild-type MCL-1 (WT) or phospho-mutant MCL-1 (4A) constructs. Ectopic expression of MCL-1 constructs was detected by Western blotting, β-Actin served as loading control. (**e**) Transfected cells were treated with 1 *μ*M GSK690 (RD cells) or 5 *μ*M GSK690 (RH30 cells) and/or 15 nM JNJ-26481585 for 72 h. Cell death was determined by flow cytometric analysis of DNA fragmentation of PI-stained nuclei (RD cells) or fluorescence-based microscope analysis of PI uptake using Hoechst 33342 and PI double staining (RH30 cells). In (**b,c,e**) mean and S.D. of three independent experiments performed in triplicate are shown; *P<0.05; **P<0.01; ***P<0.001

To further examine the involvement of the mitochondrial pathway in GSK690/JNJ-26481585-induced apoptosis, we overexpressed murine BCL-2 (mBCL-2), as confirmed by Western blotting (Figure 6a). Importantly, BCL-2 overexpression significantly diminished GSK690/JNJ-26481585-induced cell death and reduction of cell viability in RD and RH30 cells (Figures 6b and c, Supplementary Fig. 8). Also, ectopic expression of wild-type or a phospho-defective, non-degradable MCL-1 mutant (4A) (Figure 6d) significantly reduced cell death upon GSK690/JNJ-26481585 cotreatment (Figure 6e). These findings demonstrate that BCL-2 and MCL-1 inhibit GSK690/JNJ-26481585-induced cell death, emphasizing therole of the mitochondrial pathway in this model of cell death.

## Discussion

In recent years, there has been a growing interest in targeting epigenetic modifiers for cancer therapy. Overexpression of the histone demethylase LSD1 frequently occurs in cancer^19^ and high LSD1 levels were recently reported in primary RMS samples.^29, 30^ Therefore, we investigated the potential of pharmacological LSD1 inhibitors as cancer therapeutics alone and in combination with HDAC inhibitors. Here, we identify a synergism of HDAC inhibitors (i.e., JNJ-26481585, SAHA) and LSD1 inhibitors (i.e., GSK690, Ex917) to induce cell death in RMS cells. In contrast, LSD1 inhibitors as single agents proved to be insufficient for cell death induction in RMS cells, underscoring the relevance of cotargeting epigenetic modifiers for the treatment of RMS. Combined LSD1 and HDAC inhibition exhibits some tumor selectivity, as LSD1 and HDAC inhibitors show no or little cytotoxicity against murine myoblasts at concentrations that potently trigger cell death in RMS cells.

Furthermore, we provide novel insights into the molecular mechanisms underlying this synergism of concomitant LSD1 and HDAC inhibition. We show that GSK690 and JNJ-26481585 act together to induce intrinsic apoptosis by changing the ratio of pro- and antiapoptotic BCL-2 proteins in favor of apoptosis. This conclusion is supported by several independent lines of evidence.

First, GSK690 and JNJ-26481585 cooperate to transcriptionally upregulate several proapoptotic BH3-only proteins, that is, BMF, PUMA and NOXA or BIM, which is accompanied by increased protein levels. The overall shift towards proapoptotic BH3-only proteins is likely to be critical for the synergistic induction of cell death, as the portfolio of individual BH3-only proteins that is increased by GSK690/JNJ-26481585 cotreatment varies between the investigated cell lines. While the combination treatment increases BMF and PUMA expression in both RMS cell lines, BIM and NOXA are upregulated in addition to BMF in RD and RH30 cells, respectively. Upregulation of BMF, PUMA, BIM, and NOXA might well be a direct effect of GSK690/JNJ-26481585-stimulated chromatin modulation, since their mRNA levels increase already early (4 h) after cotreatment. Second, GSK690/JNJ-26481585-stimulated upregulation of proapoptotic BH3-only proteins is required for cell death induction, since individual silencing of BMF, BIM or NOXA significantly reduces GSK690/JNJ-26481585-mediated cell death. The fact that GSK690/JNJ-26481585-induced cell death does not depend on a single BH3-only protein further underlines the importance of the overall shift towards proapoptotic BH3-only proteins. Third, knockdown of BAK as a direct regulator of MOMP significantly protects from GSK690/JNJ-26481585-induced cell death. Fourth, overexpression of antiapoptotic BCL-2 or MCL-1 significantly reduces GSK690/JNJ-26481585-induced cell death. This confirms the involvement of the mitochondrial pathway as both, silencing of proapoptotic as well as overexpression of antiapoptotic BCL-2 family proteins, protect from GSK690/JNJ-26481585-induced cell death. Fifth, we show that caspase activation represents an important element of cell death execution. GSK690 and JNJ-26481585 act in concert to stimulate cleavage of caspase-9 and -3, two caspases involved in mitochondria-mediated apoptosis, as well as PARP cleavage, a typical substrate of caspase-3. Caspase-dependent apoptosis is supported by rescue experiments showing that the pan-caspase inhibitor zVAD.fmk significantly reduces GSK690/JNJ-26481585-induced cell death.

Synergistic induction of cell death by LSD1 and HDAC inhibitors has previously been described for glioblastoma,^32, 33^ AML^34^ and breast cancer.^35, 36^ Our study not only reveals a synergism of concomitant LSD1 and HDAC inhibition in RMS, but – more importantly – elucidates the molecular mechanisms underlying this synergistic induction of cell death. We show for the first time that GSK690/JNJ-26481585 cotreatment induces intrinsic apoptosis by shifting the balance towards proapoptotic BCL-2 proteins. While the combination of LSD1 knockdown and SAHA treatment has previously been reported to downregulate BCL-2 expression and to increase BAX levels, the functional relevance of these changes for the combination-induced cell death has, however, remained obscure.^36^ Furthermore, in the present study we demonstrate synergistic tumor cell death using specific LSD1 inhibitors such as GSK690 and Ex917, whereas most previous combination studies were conducted with Tranylcypromine or Pargyline,^32, 33, 35, 36^ which are unspecific LSD1 inhibitors that mainly target MAO-A and/or MAO-B.^26^

HDAC inhibitors as single agents have previously been reported to engage intrinsic apoptosis by increasing proapoptotic proteins.^37, 38^ In RMS, we previously demonstrated that JNJ-26481585 as single agent at cytotoxic concentrations exerts potent antitumor activity *in vitro* and *in vivo* by engaging mitochondrial apoptosis.^39^ Also, we showed that subtoxic doses of JNJ-26481585 sensitize RMS cells towards several common chemotherapeutics by enhancing apoptosis via the intrinsic pathway.^40^ In the present study, we show that combined inhibition of histone demethylation and deacetylation upregulates proapoptotic BH3-only proteins, indicating that histone demethylation by LSD1 acts in concert with histone deacetylation by HDACs to repress active transcription in RMS cells. Consistently, LSD1 is part of the corepressor complex CoREST, containing also HDAC1 and 2, which augment the gene repressor activity of LSD1.^13, 14, 15, 16^

Our study highlights LSD1 as an epigenetic drug target for RMS. While high LSD1 levels have previously been described in patient-derived RMS samples,^29, 30^ the functional relevance of LSD1 as a therapeutic target in RMS has remained largely unknown. By demonstrating that specific LSD1 inhibitors synergize with HDAC inhibitors to induce RMS cell death, our study has important implications for the development of novel approaches for epigenetic therapy of RMS. Thus, concomitant targeting of epigenetic modifiers using LSD1 and HDAC inhibitors represents a promising new strategy for the treatment of RMS that warrants further investigation.

## Materials and methods

### Cell culture and chemicals

RD, RMS13, TE381.T, and C2C12 cell lines were obtained from the American Type Culture Collection (ATCC) (Manassas, VA, USA), RH30 cells from the Deutsche Sammlung von Mikroorganismen und Zellkulturen GmbH (Braunschweig, Germany). Cells were maintained in RPMI 1640 or DMEM GlutaMAXX medium (Life Technologies Inc., Eggenstein, Germany) supplemented with 10% fetal calf serum (FCS), 1% Penicillin/Streptomycin and 1 mM sodium pyruvate (Invitrogen, Heidelberg, Germany). The caspase inhibitor zVAD.fmk was purchased from Bachem (Heidelberg, Germany), Necrostatin-1 from Biomol (Hamburg, Germany), JNJ-26481585 and SAHA (Vorinostat) from Selleck Chemicals (Houston, TX, USA). LSD1 inhibitors GSK690^41, 42^ and Ex0000917 were kindly provided by GlaxoSmithKline and Roche, respectively. Chemicals were purchased from Sigma-Aldrich (Taufkirchen, Germany) or Carl Roth (Karlsruhe, Germany) unless otherwise indicated.

### Determination of cell viability and cell death

Cell viability was assessed by MTT (3-(4,5-dimethylthiazol-2-yl)-2,5-diphenyltetrazolium bromide) assay according to the manufacturer's instructions (Roche Diagnostics, Mannheim, Germany). Cell death was measured by flow cytometric analysis (FACS Canto II, BD Biosciences, Heidelberg, Germany) of DNA fragmentation of PI-stained nuclei as described before^43^ or by fluorescence-based microscope analysis of PI uptake using Hoechst 33342 and PI double staining (both Sigma-Aldrich) and ImageXpress Micro XLS Widefield High-Content Analysis System and MetaXpress Software according to the manufacturer's instructions (Molecular Devices Sunnyvale, CA, USA).

### Transduction

For BCL-2 overexpression, Phoenix packaging cells were transfected with 20 *μ*g of murine stem cell virus (pMSCV, Clontech, Mountain View, CA, USA) vector containing murine BCL-2 (mBCL-2) or EV using calcium phosphate transfection as described previously.^39^ Stable cell lines were generated by lentiviral transduction and selected with 10 *μ*g/ml Blasticidin (Invitrogen). For MCL-1 overexpression, cells were transfected with 20 *μ*g of pCMV-Tag3B plasmids, kindly provided by Genentech Inc. (South San Francisco, CA, USA), containing EV, wild-type MCL-1 (WT), or phospho-mutant MCL-1 (4A) using Lipofectamine 2000 (Life Technologies, Inc.) and selected with 0.5 mg/ml G418 (Carl Roth).

### RNA interference

For transient knockdown by siRNA, cells were reversely transfected with 10 nM (BMF, BIM, NOXA) and 20 nM (BAK) SilencerSelect siRNA (LifeTechnologies) using Lipofectamine RNAiMAX reagent and OptiMEM (both Life Technologies, Inc.). Following siRNA constructs were used: control siRNA (4390842) and targeting siRNAs (s40385 and s40387 for BMF, s195012 and s223065 for BIM, s10708 and s10709 for NOXA, s1880 and s1881 for BAK).

### Western blot analysis

Western blot analysis was performed as previously described^43^ using the following antibodies: mouse anti-NOXA, rat anti-BMF, rabbit anti-MCL-1 (Enzo Life Science, Farmingdale, NY, USA), mouse anti-BCL-2, rabbit anti-BAK (BD Biosciences), rabbit anti-caspase-3, rabbit anti-caspase-9, rabbit anti-BIM, mouse anti-PARP, rabbit-anti PUMA, rabbit-anti BCL-x_L_ (Cell Signaling, Beverly, MA, USA), mouse anti-GAPDH (HyTest, Turku, Finland), rabbit anti-H3K4me2 (Diagenode, Liège, Belgium), rabbit anti-acetylated histone H3 (Merck Millipore, Darmstadt, Germany) and mouse anti-histone H3 (Abcam) or mouse anti-*β*-Actin (Sigma, Germany). Goat anti-mouse, goat anti-rabbit and goat anti-rat IgG conjugated to horseradish peroxidase (Santa Cruz Biotechnology, Santa Cruz, CA, USA) and enhanced chemiluminescence (Amersham Biosciences, Freiburg, Germany) or infrared dye-labeled secondary antibodies and infrared imaging (Odysee Imaging System, LI-COR Biosciences, Bad Homburg, Germany) were used for detection. For detection of histone modifications cells were lysed using RIPA buffer supplemented with Pierce Nuclease (Thermo Fisher, Waltham, MA, USA). Representative blots of at least two independent experiments are shown.

### Quantitative real-time PCR

Total RNA was isolated by using peqGOLD Total RNA kit (Peqlab, Erlangen, Germany) according to the manufacturer's instructions. For cDNA-synthesis, 1 *μ*g of total RNA was used to synthesize the corresponding cDNA using the RevertAid H Minus First Strand cDNA Synthesis Kit (MBI Fermentas GmbH, St. Leon-Rot, Germany) according to the manufacturer's protocol with the use of the random primers. For quantification of gene expression levels, SYBR-green based quantitative real-time PCR (Applied Biosystems, Darmstadt, Germany) was performed using the 7900GR fast real-time PCR system (Applied Biosystems). Data were normalized on 28S-rRNA expression as a reference. Analysis of the melting curves served as control for the specificity of the amplified products. Relative expression levels of the target transcript were calculated compared to the reference transcript by using the ΔΔc_t_-method. At least three independent experiments in duplicate were performed for each gene. All primers were purchased by Eurofins (Hamburg, Germany) and are listed in Supplementary Table 2.

### Statistical analysis

Statistical significance was calculated using Student's t-test (two-tailed, two samples, equal variance). Drug interaction was analyzed by the CI method using CalcuSyn software (Biosoft, Cambridge, UK);^44^ CI<0.9 indicates synergism, 0.9–1.1 additivity and CI>1.1 antagonism.
