# Supplementary material for: Concomitant epigenetic targeting of LSD1 and HDAC synergistically induces mitochondrial apoptosis in rhabdomyosarcoma cells
Source: Cell Death Dis. 2017 Jun 15;8(6):e2879–. doi: 10.1038/cddis.2017.239 (PMC5520898; doi:10.1038/cddis.2017.239)
Supplement: Supplementary Material [file cddis2017239x1.pdf]

Supplementary Figure 1

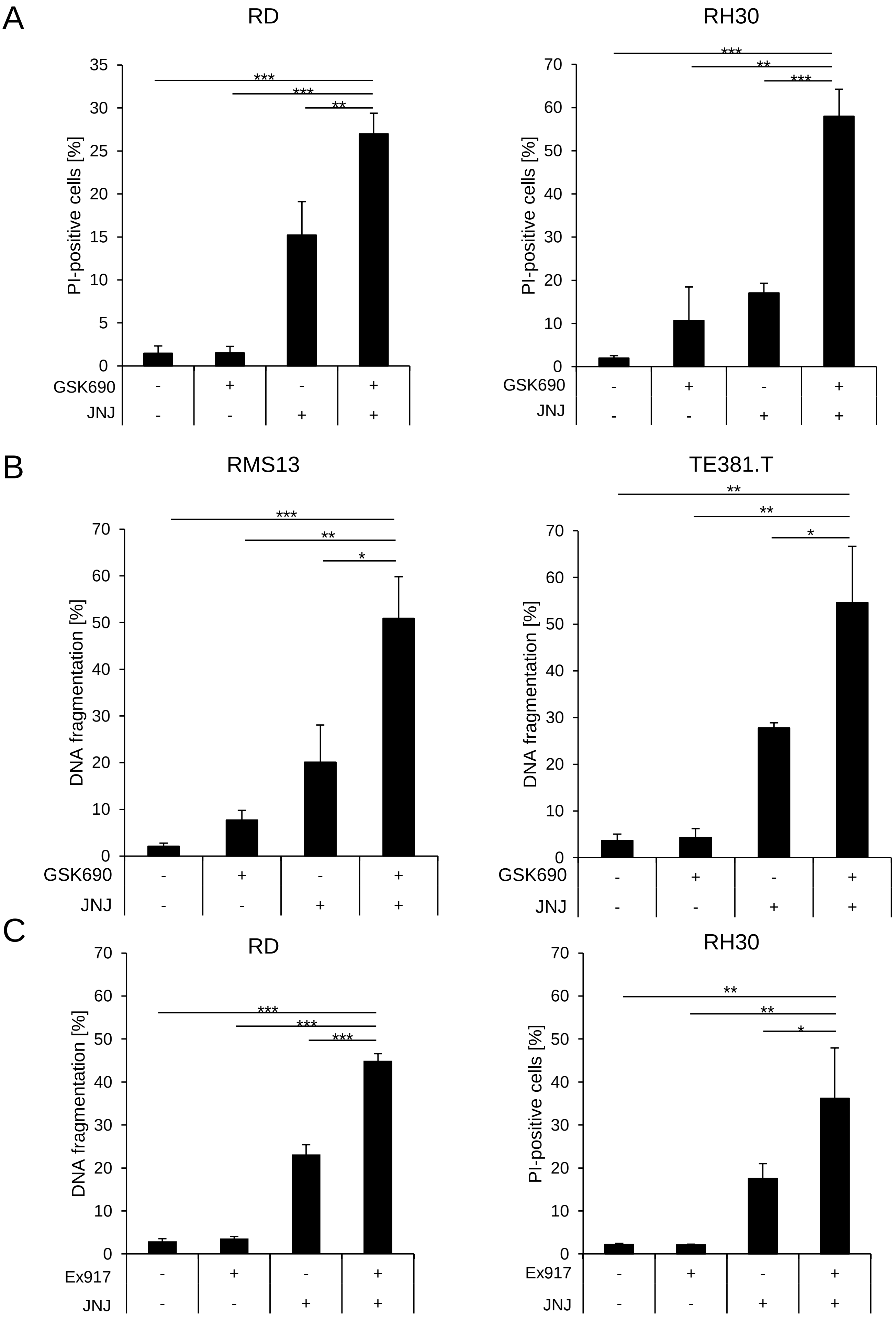

Supplementary Figure 1

D

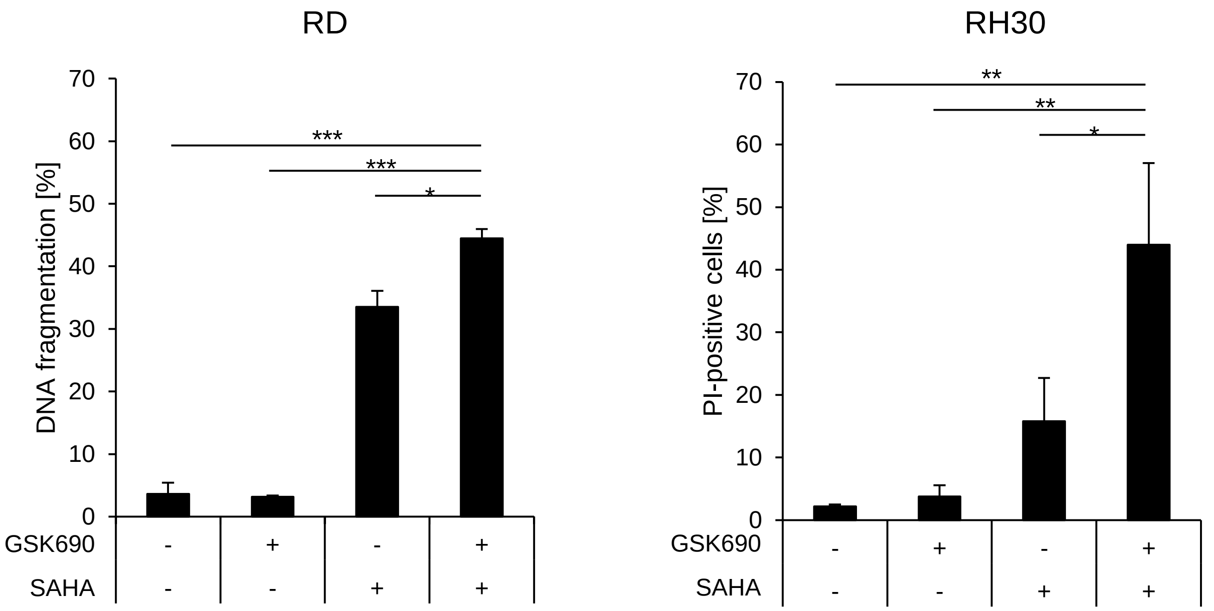

E

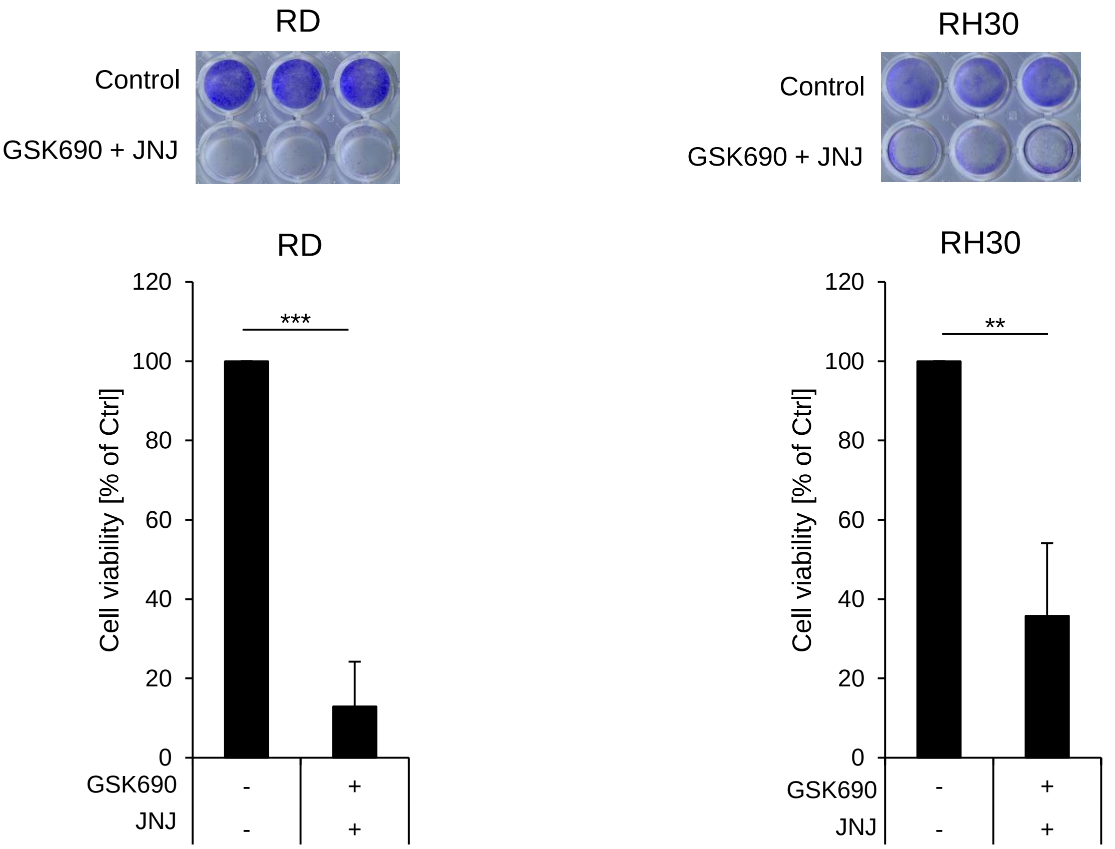

Supplementary Figure 2

A

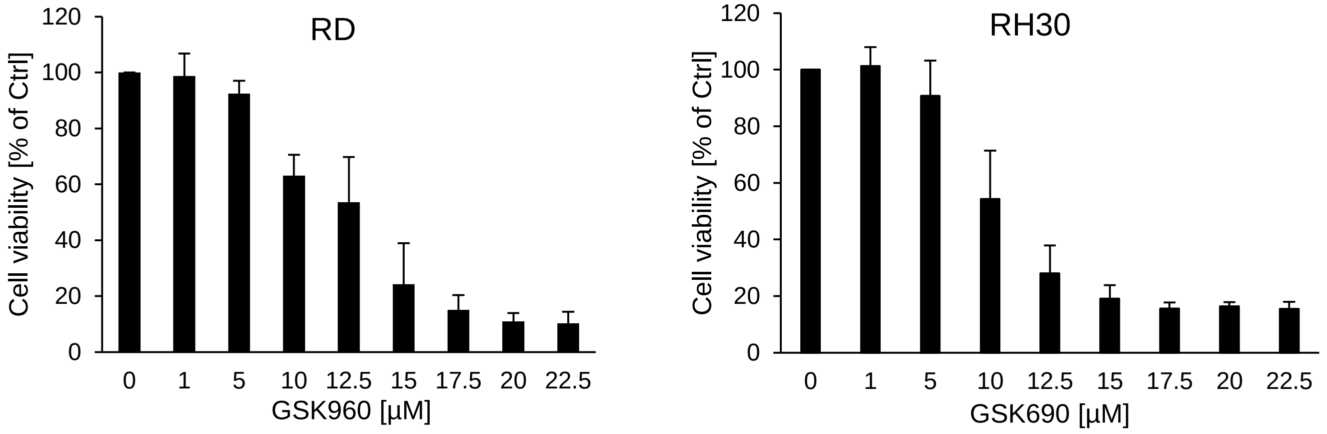

B

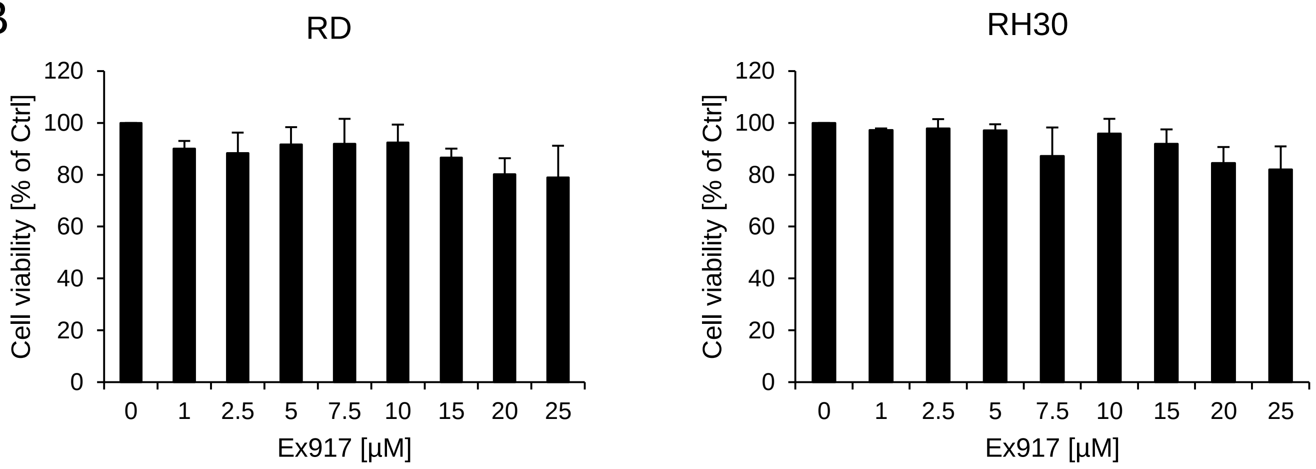

Supplementary Figure 3

A

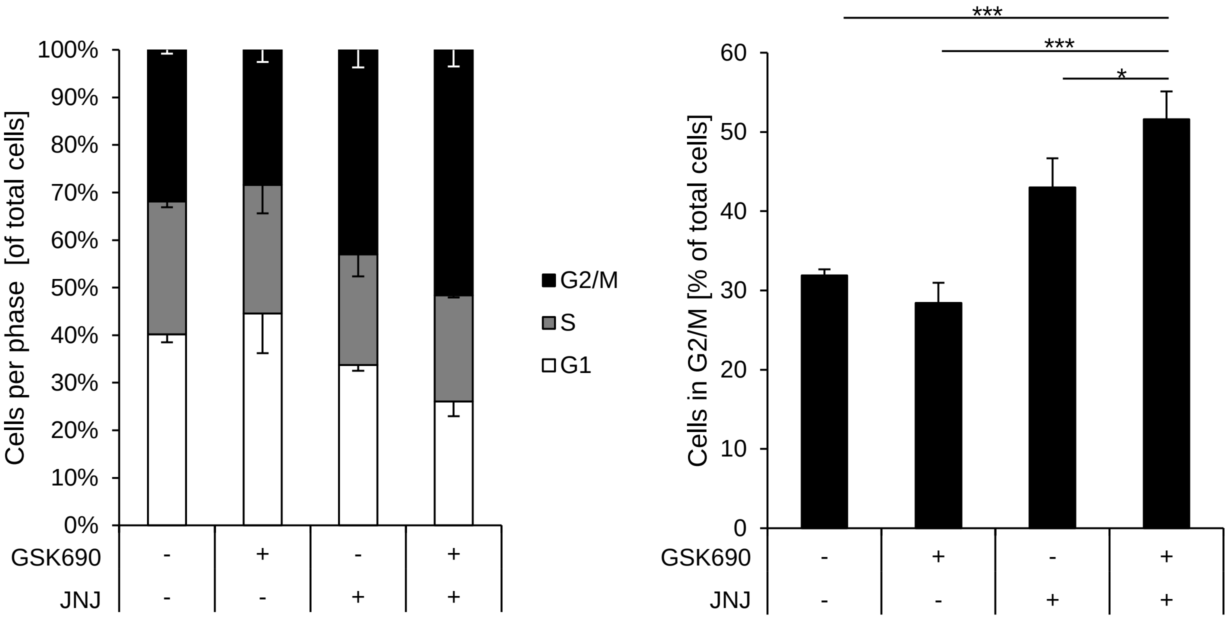

B

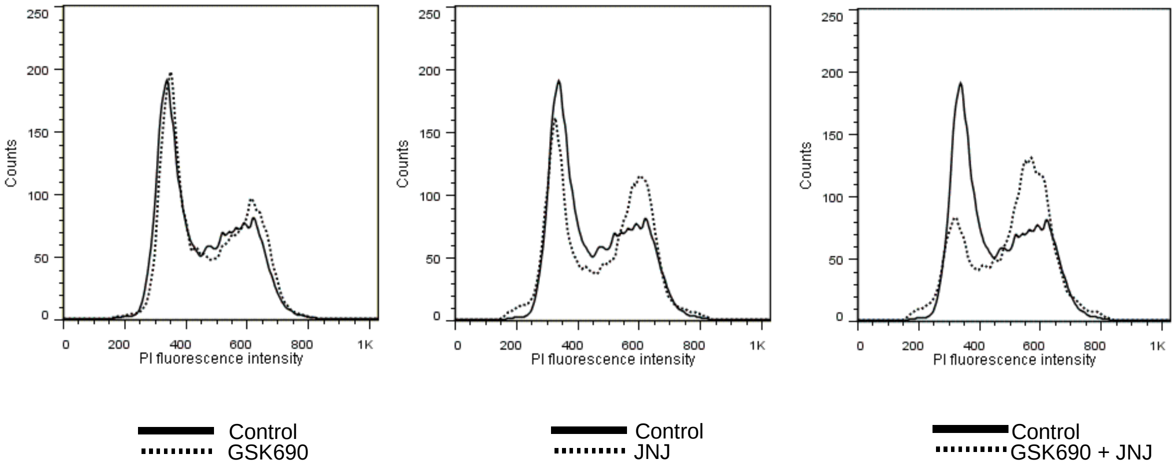

Supplementary Figure 4

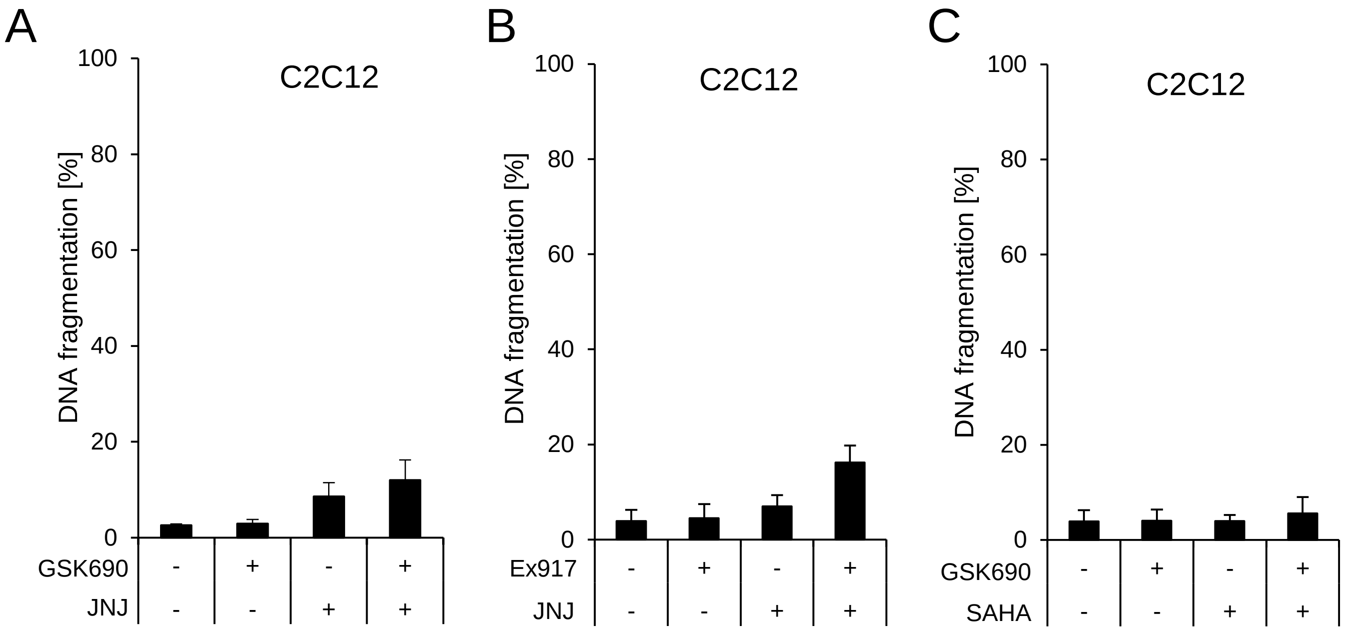

Supplementary Figure 5

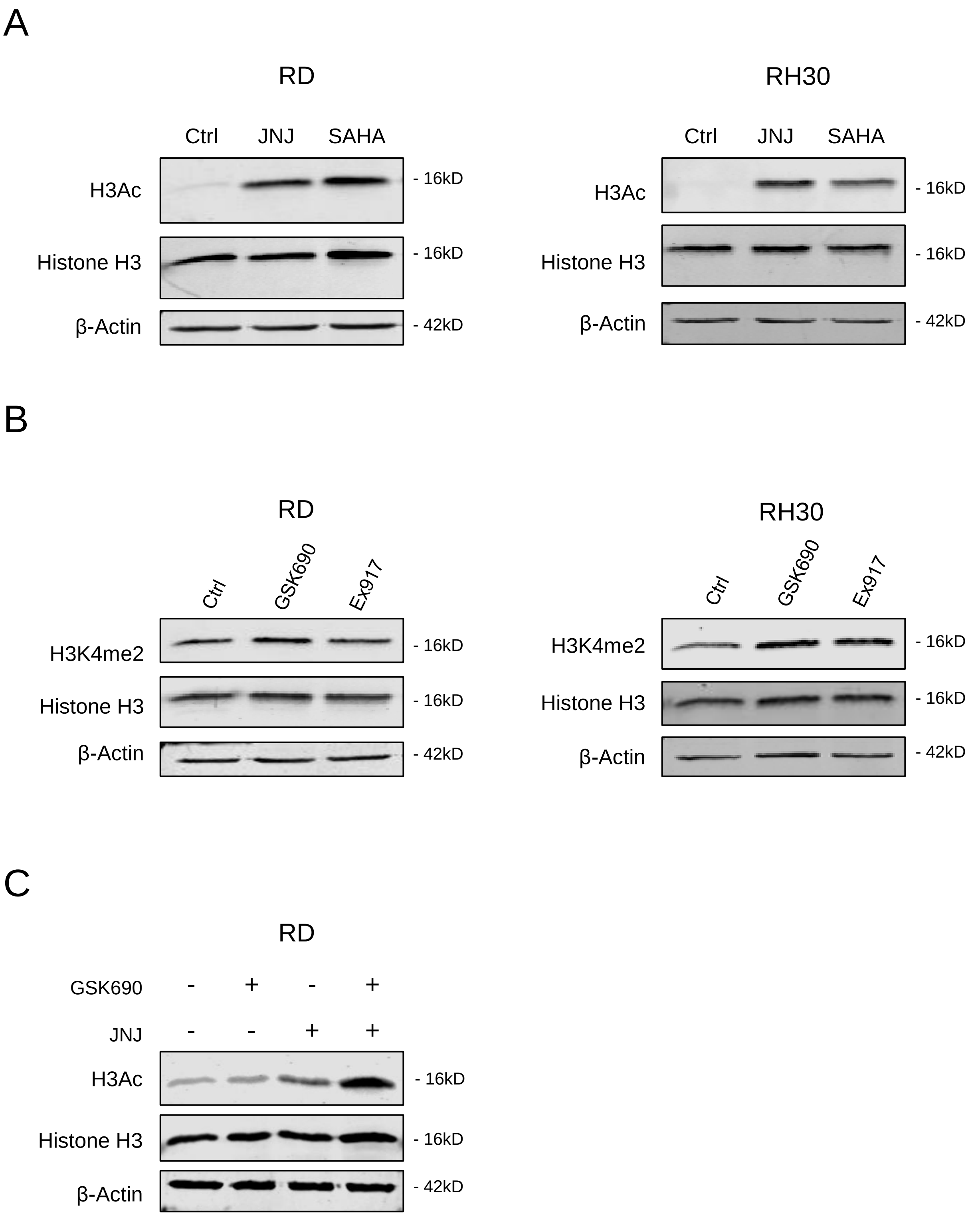

Supplementary Figure 6

A

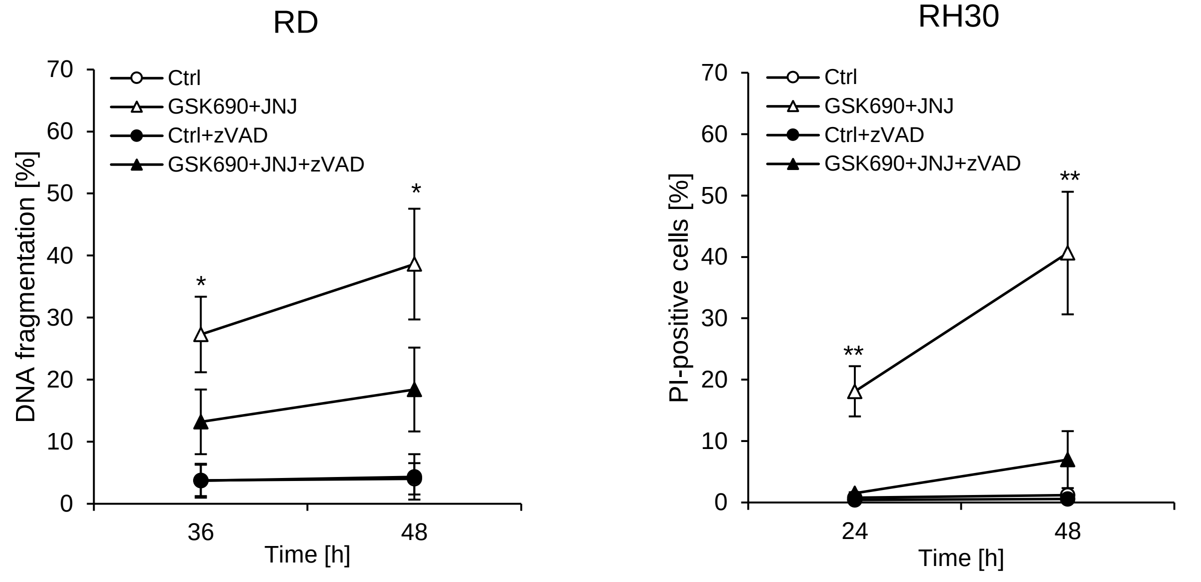

B

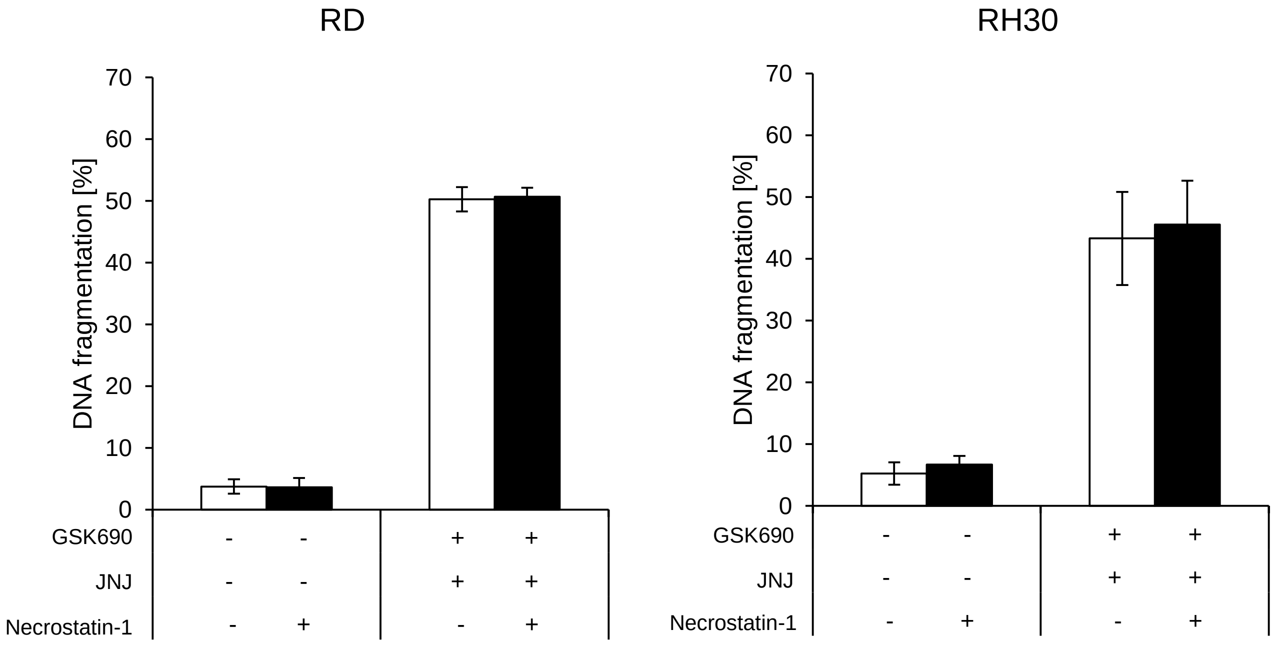

Supplementary Figure 7

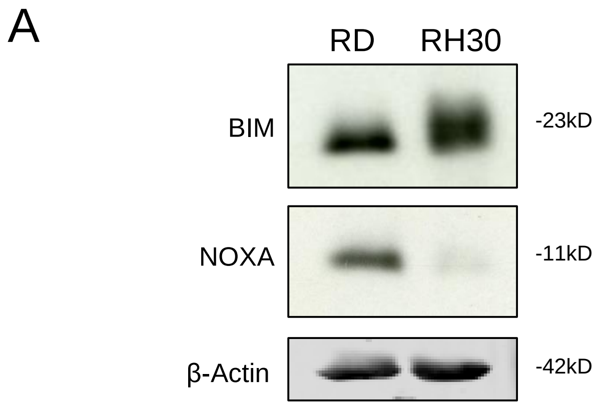

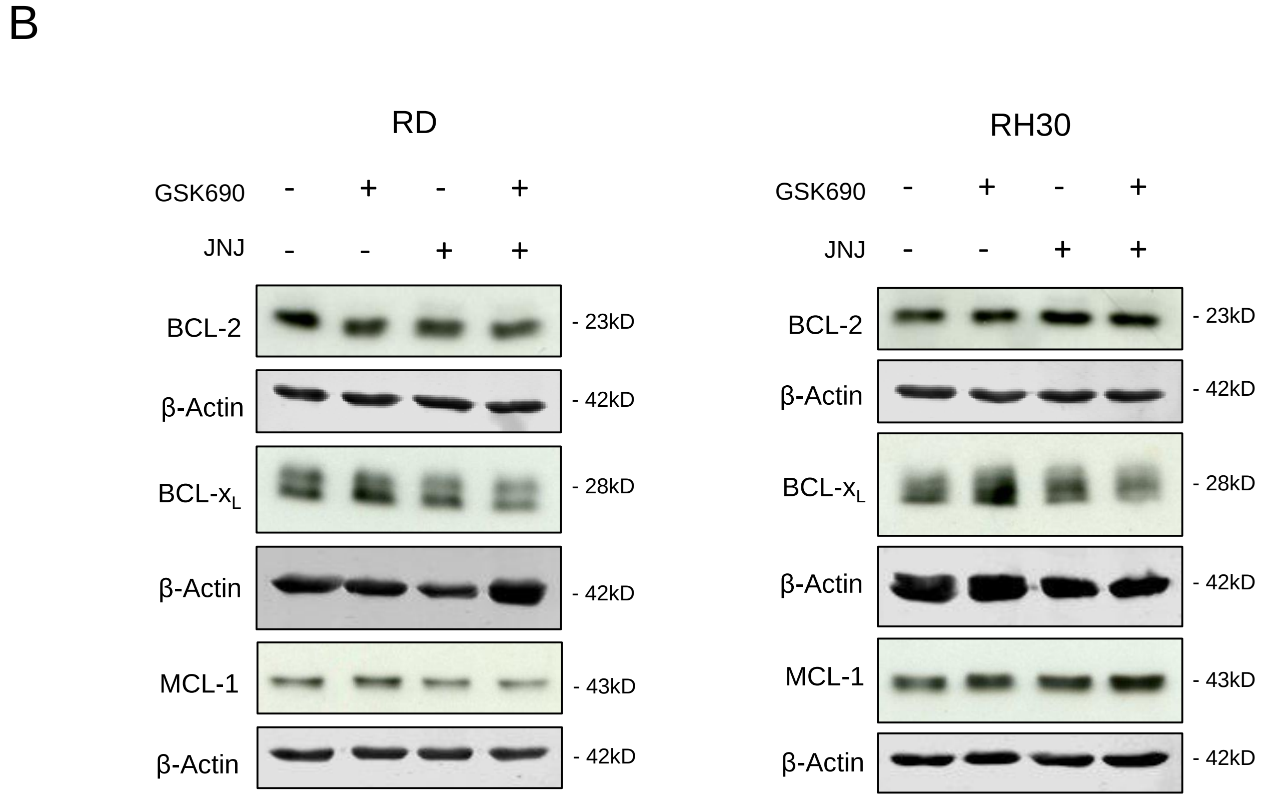

Supplementary Figure 8

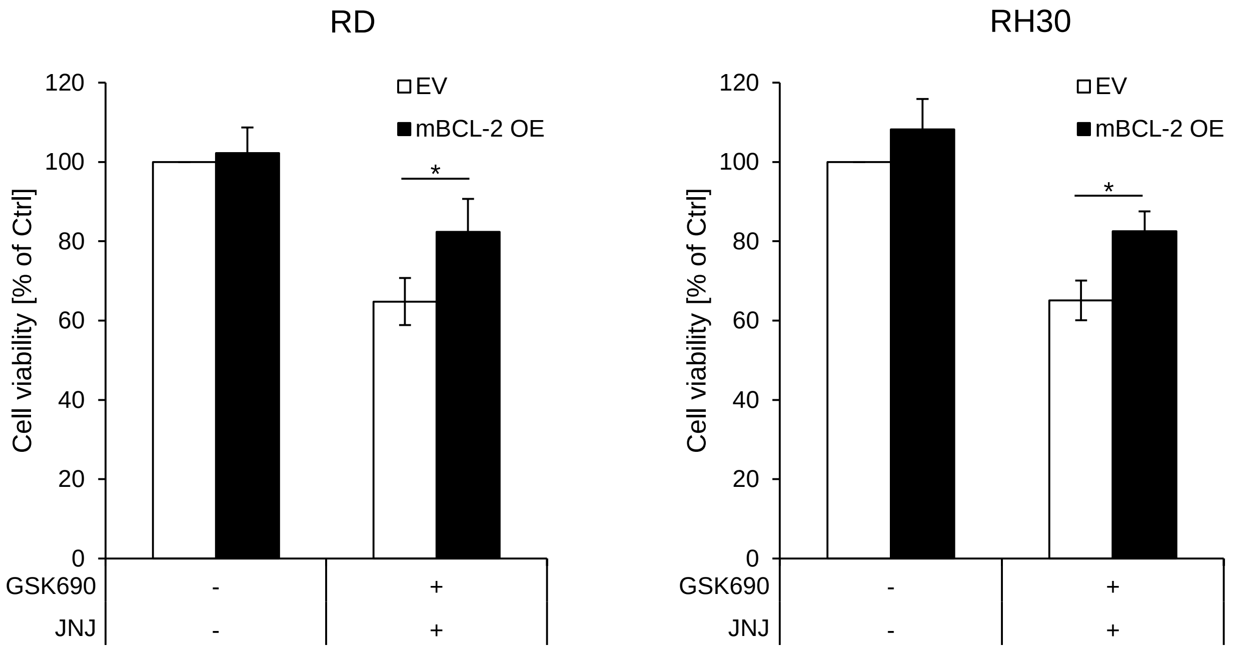

Supplementary Figure Legends

Supplementary Figure 1: GSK690/SAHA combination and Ex917/JNJ-26481585 combination induce cell death in RMS cell lines

A RD cells were treated with 1 µM GSK690 and/or 15 nM JNJ-26481585 and RH30 cells were treated with 10 µM GSK690 and/or 15 nM JNJ-26481585 for 72 hours. Cell death was measured by fluorescence-based microscope analysis of PI uptake using Hoechst 33342 and PI double-staining. **B,** Cells were treated with 10 µM GSK690 (RMS13) or 1 µM GSK690 (TE381.T) and/or 15 nM JNJ-26481585 for 72 hours. Cell death was determined by flow cytometric analysis of DNA fragmentation of PI-stained nuclei. **C and D,** Cells were treated for 72 hours with 10 µM Ex917 and/or 15 nM JNJ-26481585 (C) or 1 µM GSK690 (RD cells) or 10 µM GSK690 (RH30 cells) and/or 2 µM SAHA (D). Cell death was determined by flow cytometric analysis of DNA fragmentation of PI-stained nuclei (RD cells) or fluorescence-based microscope analysis of PI uptake using Hoechst 33342 and PI double-staining (RH30 cells). **E,** Cells were treated with 1 µM GSK690 (RD) or 10 µM GSK690 (RH30) and 15 nM JNJ-26481585 for 120 hours and cell viability was determined by crystal violet assay. In **A-E**, mean and SD of three independent experiments performed in triplicate are shown; *$P<0.05$; **$P<0.01$; ***$P<0.001$.

Supplementary Figure 2: GSK690 reduces cell viability

A and B RD and RH30 cells were treated with indicated concentrations of GSK690 or Ex917 for 72 hours. Cell viability was determined by MTT assay. In **A and B**, mean and SD of three independent experiments performed in triplicate are shown.

Supplementary Figure 3: GSK690/JNJ-26481585 cotreatment arrests cells in G2/M phase

RD cells were treated with 1 µM GSK690 and/or 15 nM JNJ-26481585 for 24 hours. DNA content of fixed and PI-stained nuclei was determined by flow cytometry and analyzed with FlowJo software. For **A,** Mean and SD of three independent experiments performed in triplicate are shown; *P<0.05; **P<0.01; ***P<0.001. In **B,** the histogram of an exemplarily experiment is shown.

Supplementary Figure 4: GSK690/JNJ-26481585 combination treatment does not affect non-malignant C2C12 myoblasts

A-C, C2C12 myoblast cells were treated 1 µM GSK690 and/or 15 nM JNJ-26481585 (A), 10 µM Ex917 and/or 15 nM JNJ-26481585 (B) or 1 µM GSK690 and/or 2 µM SAHA (C) for 72 hours. Cell death was measured by flow cytometric analysis of DNA fragmentation of PI-stained nuclei. In **A-C**, mean and SD of three independent experiments performed in triplicate are shown.

Supplementary Figure 5: LSD1 and HDAC inhibitors are target specific for the respective histone modification

A and B, Cells were treated with HDAC (15 nM JNJ-26481585; 2 µM SAHA) and LSD1 inhibitors (1 µM GSK690 (RD); 10 µM GSK690 (RH30); 10 µM Ex917) for 3 hours. Histone acetylation and H3K4 dimethylation were detected by Western blotting. Histone H3 and β-Actin served as loading controls. **C,** RD Cells were treated with 1 µM GSK690 and/or 15 nM JNJ-26481585 for 1 hour. Histone acetylation was detected by Western blotting. Histone H3 and β-Actin served as loading controls.

Supplementary Figure 6: Rescue with zVAD.fmk at early time points for GSK690/JNJ-26481585 combination treatment

A Cells were treated for indicated time points with 1 µM GSK690 (RD) or 10 µM GSK690 (RH30) and/or 15 nM JNJ-26481585 in the presence or absence of 50 µM zVAD.fmk. Cell death was measured by flow cytometric analysis of DNA fragmentation of PI-stained nuclei (RD) or by fluorescence-based microscope analysis of PI uptake using Hoechst 33342 and PI double-staining (RH30). **B,** Cells were treated for 72 hours with 1 µM GSK690 (RD) or 10 µM GSK690 (RH30) and 15 nM JNJ-26481585 in the presence or absence of 50 µM Necrostatin-1. Cell death was measured by flow cytometric analysis of DNA fragmentation of PI-stained nuclei. In **A and B**, mean and SD of three independent experiments performed in triplicate are shown; *P<0.05; **P<0.01; ***P<0.001.

Supplementary Figure 7: Expression levels of BCL-2 proteins in RMS cells

A, Constitutive protein levels of NOXA and BIM of untreated RD and RH30 cells were assessed by Western blotting, β-Actin was used as loading control. **B,** Cells were treated with 1 µM GSK690 (RD) or 10 µM GSK690 (RH30) and/or 15 nM JNJ-26481585 for 21 hours (RD) and 15 hours (RH30). Protein levels of BCL-2, BCL-x_L and MCL-1 were detected by Western blotting, β-Actin served as loading control.

Supplementary Figure 8: Overexpression of BCL-2 partially rescues GSK690/JNJ-26481585-induced reduction of cell viability

Cells were treated with 1 µM GSK690 (RD) or 10 µM GSK690 (RH30) and/or 15 nM JNJ-26481585 for 36 hours (RD) and 24 hours (RH30). Cell viability was determined with crystal violet assay. Mean and SD of three independent experiments performed in triplicate are shown; *P<0.05; **P<0.01; ***P<0.001.

Supplementary Materials and Methods

Determination of cell viability

For crystal violet staining cells were stained for 10 minutes in crystal violet solution (0.5% crystal violet, 30% ethanol, and 3% formaldehyde), washed with tap water and air-dried. For colorimetric measurement crystal violet was resolved in 1% SDS and absorbance at 550 nM was quantified by microplate reader (Infinite M100, Tecan, Männedorf, Switzerland).

Cell cycle analysis

DNA content of fixed and PI-stained nuclei was determined by flow cytometry and analyzed by FlowJo software (Tree Star Inc., Ashland, OR, USA) according to the manufacturer's instructions.

Supplementary Table T1: Synergistic induction of cell death by GSK690 and JNJ-26481585

RD		JNJ-26481585 [nM]		
		5	10	15
GSK690 [µM]	1	0.455	0.516	0.263
	5	0.708	0.47	0.254
	10	0.33	0.232	0.253

RH30		JNJ-26481585 [nM]		
		5	10	15
GSK690 [µM]	1	0.923	0.871	0.775
	5	0.751	0.546	0.636
	10	0.332	0.362	0.454

Combination index was calculated by CalcuSyn software as described in Materials and Methods for data on GSK690- and/or JNJ-26481585-induced cell death as shown in Fig. 1A; CI<0.9 indicates synergism, 0.9-1.1 additivity and CI>1.1 antagonism.

Supplementary Table 2: List of Primers

Target	Forward Primer (5'-3')	Reverse Primer (5'-3')
28S	TTGAAAATCCGGGGGAGAG	ACATTGTTCCAACATGCCAG
NOXA	GGAGATGCCTGGGAAGAAG	CCTGAGTTGAGTAGCACACTCG
BIM	CATCGCGGTATTCGGTTC	GCTTTGCCATTTGGTCTTTTT
BMF	GAGACTCTCTCCTGGAGTCACC	CTGGTTGGAACACATCATCCT
PUMA	GACCTCAACGCACAGTACGA	GAGATTGTACAGGACCCTCCA
